# Is task-shifting a solution to the health workers’ shortage in Northern Ghana?

**DOI:** 10.1371/journal.pone.0174631

**Published:** 2017-03-30

**Authors:** Eunice Okyere, Lillian Mwanri, Paul Ward

**Affiliations:** 1 Social Science Group, Kintampo Health Research Centre, Kintampo, Ghana Health Service, Ghana; 2 Discipline of Public Health, Faculty of Medicine, Nursing and Health Sciences, Flinders University, Adelaide, Australia; University of South Australia, AUSTRALIA

## Abstract

**Objective:**

To explore the experiences and perceptions of health workers and implementers of task-shifting in rural health facilities in Upper East Region, Ghana.

**Methods:**

Data was collected through field interviews. A total of sixty eight (68) in-depth interviews were conducted with health workers’ in primary health care facilities (health centres); Four in-depth interviews with key persons involved in staff management was conducted to understand how task-shifting is organised including its strengths and challenges. The health workers interview guide was designed with the aim of getting data on official tasks of health workers, additional tasks assigned to them, how they perceive these tasks, and the challenges associated with the practice of task-shifting.

**Findings:**

Task-shifting is a practice being used across the health facilities in the study area to help reduce the impact of insufficient health workers. Generally, health workers had a comprehensive training that supported the organisation of task-shifting. However, staff members’ are sometimes engaged in tasks above their level of training and beyond their actual job descriptions. Adequate training is usually not provided before additional tasks are assigned to staff members. Whilst some health workers perceived the additional tasks they performed as an opportunity to learn new skills, others described these as stressful and overburdening.

**Conclusion:**

Task-shifting has the potential to contribute to addressing the insufficient health workforce, and thereby improving health delivery system where the procedures are well defined and staff members work in a coordinated and organised manner. The provision of adequate training and supervision for health workers is important in order to improve their expertise before additional tasks are assigned to them so that the quality of care would not be compromised.

## Introduction

Human resources remain an essential component of the global health systems (GHS), and its impact is determined by the availability of, access to and the provision of quality health care delivery by health workers [1,2]. Adequate health care workers, however, remain a major challenge for African countries. Although Africa is made up of about 11% of the world’s population (with consistently high mortality rates), the continent accounts for about 25% of the global burden of diseases—with only 4% of the global health workforce to tackle this problem [3]. Countries such as Ghana, Nigeria, Zambia and South African, whose health care systems are considered better off within the continent, have already over-stretched their health systems due, in part, to health workers shortage in rural areas [4]. In these countries, less than 50% of the required personnel are available to serve the rural populations (in inaccessible remote villages, and in densely populated village and slum areas). To salvage the situation, under qualified health personnel are allowed to provide alternative health care within their community [1,5].

As such, health workers shortage can hinder access to quality healthcare and the impact is greater if such shortages are accompanied by an unequal distribution of the work force [1,6]. Apart from the loss of health workers in rural areas leading to serious accessibility problems [7], and comparatively high rate of mortality in these areas [8–10], lower nurse-to-patient ratio has also led to more complications and poorer health outcomes for patients [11]. It has also led to increased cost and overcrowding by rural inhabitants in urban hospitals [9,12,13]. Increased workload brings about low motivation and work-related stress [14]. The World Health Organization (WHO) in responding to the perceived interest of policy makers in various countries in addressing the health workers shortage encouraged task shifting [2].

Task-shifting makes use of already available human resource by delegating tasks requiring high skills to health workers with lower qualification [2,15]. In Ghana (a case in point for the current study) for example, medical assistants have been used to diagnose and treat various forms of illnesses (from terminal diseases to common cold) for years [16]. At certain times, complex tasks are delegated to mid-level cadres of health workers, such as non-physician clinicians and midwives as in the case of Malawi, Mozambique and Tanzania where about 90% of emergency obstetric operation, including caesarean sections are carried out by clinical officers [17,18]. Despite the use of this strategy, WHO has emphasized the need for tasks to be carefully selected, roles well defined and adequate supervision put in place [19] in the implementation of task-shifting.

Though Ghana and many other countries rely largely on task-shifting in tackling the human resource shortage especially in the rural areas [20], the level of the implementation of task-shifting and the extent to which it contributes to the amelioration of the effect of rural health workers shortage has not been thoroughly investigated and understood in Ghana. This paper aims to fill this gap in knowledge by exploring the thematic issue of rural health workers and implementers of task-shifting, based on what they say, and not what is imagined and theorized about them.

## Methods

### Ethics statement

Ethical approval for the study was obtained from the Navrongo Health Research Centre Institutional Review Board (NHRCIRB) in Ghana, and from the Flinders University’s Social and Behavioural Research Ethics Committee (Project Number 6804) in Australia. Written informed consent was obtained from the study participants prior to the start of the interview sessions.

### Study setting

The study was conducted in 10 out of the 13 districts in the Upper East Region of Ghana. These districts include Garu Tempane, Pusiga, Binduri, Builsa South, Bawku East and Bawku West, Bolgatanga, Kassena Nankana East, Kassena Nankana West and Builsa North. For the study area diversity, the 10 districts selected for the study were sampled (using a non-probability technique) to obtain a mix of health centres situated in typically rural, peri-rural and urban settings. The region was purposively selected for the study on the basis that, the population is predominantly rural (87%) [21]. The majority of the people live in rural settings; and households are grouped into extended family units or compounds which are located far from each other, yet the people depend largely on their accustomed communal life style [22].

The region has the highest levels of poverty and maternal mortality and the lowest level of supervised birth deliveries [21,23]. It is one of the deprived regions within the northernmost edge of the country; and one of the places where health workers least expect to be posted to [21]. The population is estimated to be slightly above 1million with a growth rate of 1.2%. It shares boundaries with the Republic of Burkina Faso to the north and the Republic of Togo to the East. Collectively these neighboring countries have similar demographic characteristics such as language, socio-cultural and belief systems. There is heavy movement of persons, goods and services across the various borders. This movement of persons and goods presents several challenges to disease surveillance and control particularly at the entry points. Majority of the communities in the region are not accessible at the height of the rainy season (between August and September). The national government, through the Ghana Health Service (GHS) ministry, is the major health care provider in the region.

### Data collection

Qualitative data was collected between August 2015 and January 2016. The foci were to explore the extent of the implementation of task-shifting among rural health workers: their official and additional tasks assigned to them, how they perceive these tasks, and the challenges associated with task shifting practice. A wide range of respondents working in these rural facilities were purposively selected—taking into consideration designation, ethnicity, educational levels, marital status and experience to capture their understanding (and associated impact) of task-shifting. The data collection was also informed by the secondary literature that aided in the process of content selection and verification of interview comments. The health workers interview guide (S1 File) and the health managers interview guide (S2 File) were piloted among health care workers and managers outside the study area, and therefore not part of the study to ensure that the length and structure of the interview were appropriate. All interviews were conducted in places considered convenient by study participants. They were conducted in English by the principal investigator who explored thoroughly each item on the interview guide until no new issues emerged. The interviews were tape-recorded and converted into detailed transcripts each day and supplemented with notes taken during the interview. A total of 68 in-depth interviews were conducted with the Medical Assistants (MA), midwives, General Registered Nurses (GRN), Enrolled Nurses (EN), Community Health Officers (CHO), Disease Control Officers (DCO), Psychiatric Nurses (PN), optometrist and health nurse aides who worked in the 26 health centres within the selected 10 districts which included the heads (also known as the *in-charges*) of these facilities. The heads of the various health centres were part of the study to specifically explore in detailed the mechanisms and strategies employed in the management of task-shifting at their respective health facilities. Four key interviewees’ interviews were also conducted with health administrators and other key persons involved in staff members’ management in the region. The study used phenomenological research design to describe the meaning and importance of health workers and managers experiences and perceptions of task-shifting practice [24].

### Data analysis

The transcripts were explored through multiple readings by two authors to ensure familiarity with the data and subsequently analysed independently using Ritchie and Spencer thematic framework analysis [25]. This method of analysis uses systematic approach to manage qualitative data to improve the structure and consistency of the data [26]. Transcripts were read several times and the key and essential text identified, organised and coded. Similar codes were gathered into possible themes that reflects the opinions of respondent [27]. To minimise the chances of losing themes which were relevant, recurring themes of text were identified and allocated headings that confers to the context and further coded to several key categories and subsequently, sub-headings were identified from the thematic analysis [26]. This process of systematic analysis leads to a greater transparency, rigor and validity of the data. Triangulation of the narratives of different cadres of health workers and managers at different settings contributed to the validity of the data. Data analysis included both inductive, with categories emerging merely from the data and deductive, with categories resulting from previous knowledge [28,29]. The main themes that emerged were official (main) tasks and additional workload performed by health workers, health workers and management members perceptions on task-shifting, and health system and governance factors in implementing task-shifting.

### Rigour and trustworthiness

The process involved in evaluating the quality of research is referred to as rigour and the framework used to assess rigour is trustworthiness [30,31]. The rigour and trustworthiness of this study was therefore assessed using four criteria which include credibility, transferability, dependability, and confirmability.

The credibility criterion evaluates how accurate the study findings are in relation to the experiences of the informants [32]. In this study, credibility was improved through the audio recordings of the interviews which ensured that the exact words of the respondents were captured and after transcription a sample of the transcripts were sent to the participants to be checked and information verified [33], since it was not feasible to carry out the member-checking exercise among all participants. Persistent observation during field work also improved the credibility of this study. The researcher involved in the data collection activities (EO) stayed in the field for a longer time and this enabled her to visit the health facilities to observe the events and other activities which subsequently enhanced the information that was gathered during the in-depth interviews. Apart from the collected data been reviewed by co-authors to check for consistency in the codes and identified themes, the interpretations of the study findings were supported by participants’ quotes which further added credibility to the interpretation [34].

Additionally, the triangulation strategy used in this study improved it quality. This refers to the different data sources, investigators, analysts, and different ways of collating of data or using different ways of interpreting the data [35]. This study offers range of methodological triangulation through the different methods of data collection using in-depth interviews, documentary reviews and the researcher’s reflective notes of observation during the field work. The interviews were also conducted with different categories of health workers and management members about the same issue of task-shifting practice which enhanced the triangulation process.

Secondly, transferability criterion assesses the possibility of applying the study findings to other locations [36] and as such necessary for researchers to provide detailed information on the particular location of the research to enable readers make a decision on the applicability of the study findings to other locations [37]. The researchers has provided detailed information on the study location and the sampling method used (purposive sampling) since selecting informants purposively ensures the transferability of a study [38]. However, the researchers wish to clarify that, they do not intend to generalise the findings of this study but to give detailed description of the phenomenon under study within the particular setting or context.

Another strategy for measuring the rigour and trustworthiness referred to as dependability evaluates the consistency of the study findings and as such the need for the researcher to give detailed information regarding the methods used in the collection of the data, analysis and interpretation [31]. As such, the methods used in this study have been systematically described by the authors to enable other researchers to replicate a similar study if they want to though this could be challenging due to differences in contexts and time.

Lastly, the confirmability criteria is the extent to which the study results are influence by the informants rather than the interest, perceptions or bias of the researcher and this involves the process of auditing to establish quality [34]. The auditability process which indicates the capacity of the researcher to demonstrate a clear decision trail of development of events and the rationale of the researcher over the time of the study has been established to improve not only the confirmability strategy but also of the dependability of the research [31]. In the process of going through the audit trail, the auditor might concentrate on the notes taken during field work which comprise of methodological or personal information, reflexive journal keeping, the member checking process and triangulation [37,38]. Accordingly, detailed field notes were taken during fieldwork which confirmed the auditability of the research process. Also, this research explains clearly the rationale for the selection of the research topic, research site, recruitment of study participants and gives in-depth procedures of data collections and analysis, which check auditability in determining research quality [31,39]. Finlay (2006) suggests the need to take into consideration a holistic method to establish a rigorous understanding of occurrences of events and data during qualitative research. Therefore, the researchers made an effort to maintain rigor throughout this study, from the start to developing a suitable methodological approach, choosing apposite methods for data collection, conducting theoretically informed data analysis as well as taking into consideration the ethical issues relating to the study.

## Results and discussion

### Official (main) task and additional workload performed by health workers

Generally, health workers in the study areas are supposed to perform tasks in the disciplines in which they have received official training in. Units within the healthcare systems, of say, the maternity or disease control department should be able to respond to the needs of their client population. One way of doing this, is the creation of high performance roles that demand commitment and a willingness to accept these high-performing tasks. The primary health workers should be made to understand these roles and responsibilities, and be part of the processes in developing them. Because it enables them to have a well-define benchmarks in the realisation of their tasks.

A clearly defined task for different cadres of health professionals is essential for effective health care delivery, and for nominal inter-professional cooperation. On the other hand, imprecise allocation of tasks can lead to duplicity, and inter-personal conflicts among health workers. Subsequently, the imprecision of task allocation may lead to the ineffectiveness of healthcare workers in the delivery of health services to the clients [40,41]. Apart from defining the specific responsibilities of each cadre of health workers, other different functions are performed and satisfactory outcomes are reached when professional roles are clearly allocated. These include; guaranteeing adequate execution of each cadre’s tasks to optimising expert choice of practice to ensure that patient are efficiently managed [42].

In view of this, the Ghana Health Service (GHS) has different cadres of health workers who are officially trained in various health institutions to perform specific roles with the ultimate aim of providing quality care to the population. From a theoretical standpoint, the concept of efficiency in clarity of tasks allocation begins from the time of training. In that, the worker becomes aware of what he/she can or cannot do; what he/she should be seen to be doing when he/she begins work. As such, situating this within the GHS, all health workers (ranging from the Physician Assistants (PA) to Health Records Officers (HRO) and Health Aides (HA)) in the study area are generally performing tasks in the areas where they have officially received specialised training in. As will be explained later, this does not mean that they are not performing tasks beyond their stated terms of reference/duties that are defined in their official appointment letters. This was observed among all the different cadres of workers and across all the health facilities in the various districts. For instance, Disease Control Officers (DCOs) performed disease surveillance and control; the consultation of the sick by Physician Assistants (PA) and the delivery of babies were performed by well-trained midwives. Respondents described their official duties as follows:

*“You know as a trained Disease Control Officer [DCO], disease surveillance and control are my routine activities in this facility. I collect daily surveillance information on diseases, collate them together and report to the district health management team. Sometimes I do immunization activities and manage the cold room where the vaccines are stored”* (DCO 1)

*“Conducting deliveries is the major thing I do in this health centre. The women after delivering come for postnatal care so I check them and their babies to be sure there is nothing wrong with them. Am here to see to it that the women and their babies are healthy but you know sometimes some of these women are troublesome. They don’t even come for check-up after delivery”* (Midwife 1)

Some of these health workers were formerly employed through the National Youth Employment Program (NYEP). This was a program instituted by the GHS to help address the chronic shortage of health workers in the country. The targeted individuals for this program were those who have completed senior high school. They were given six (6) months basic training in health care to enable them to assist the health workers in the various facilities across the country. Through this short training some of the individuals succeeded in building their skills in the performance of various health related disciplines and also additional health workers were posted to remote areas to assist in health delivery. However, due to financial constraints, amid lack of a commitment to continue experimentation of the program, it was suspended. Recalling how this program benefited him, and, in tandem, the GHS, a Health Records Officer (HRO 2), explains that, the program offered him a career promotion. This is what he has to say:

*“I was first employed by the national youth employment program so I started from that level which was 2006 as an extension officer but in 2009, the program was cut off so I went for the health records course at Kintampo rural health training school. After the course I was brought back here to continue my work. So now I am a health records officer and what I do here is that when patients report to this facility for treatment, I remove their folders and record their information before they are seen by the MA [i.e. Medical Assistant, who are sometimes called physician assistants] or a nurse. After that I make sure the folders are filed properly though we don’t have a proper place for filling the folders”* (HRO 2).

The physician/medical assistants are mainly trained to consult in these rural health facilities. In most instances they are placed in managerial positions to manage the activities of various facilities, including the Community-based Health and Planning Services (CHPS) compounds within their localities. They perform supervisory roles, address challenges faced by staff members and when necessary report to the municipal directorate. Due to the management responsibilities assigned to them, they are referred to as the ‘in-charge’, which literally means, he/she is in-charge of the facility. Until recently, when the GHS introduced direct entry from the senior high school into physician assistants’ courses, these cadres of health workers were experienced nurses, with many years of work experience—some of these experienced nurses even hold a number of advance level academic qualifications. Physician Assistants (PA) 5 and 6 explained their main tasks, thus:

*“My main task here is to render primary health care services. Apart from daily general OPD [outpatient department] consultations, I manage emergencies and minor procedures like seizures and assist in the Reproductive and Child Health maternity unit. As the physician Assistant [PA] sub-municipal leader in-charge of the facility, I do supervision to ensure that all staff report to work on time, and everybody is at work in their designated units. I do monitoring too by visiting the Community Health Planning Services compound on weekly bases to check on progress of their on-going activities, and find out about their challenges so I can liaise up with the municipal directorate. I am still the liaison officer between the sub-municipal office and the municipal office”. (PA 5 in- charge of facility X–* *50years)*

*“As the PA in-charge, I am in-charge of the sub-district activities and the sub-districts are made up of the CHPS compounds and CHPS zones plus the health centre itself so I render both public health and clinical activities. In-terms of the public health I make sure that the logistics and human resources are available to be able to render those services. Basically I make sure that all the activities in the sub-districts are carried out and the reports are submitted to the districts and regional. In-terms of the clinical too I am supposed to take care of the cases that come here, I mean taking care of the sick. I do a lot here”. (PA 6, in-charge of facility K* *-31years)*

To explain this further, it was discovered during field research that, in a situation where Health Service Administrators (HSAs) are present in these facilities, they (the PAs) are also very visible in the performance of the duties of the HSAs. During the in-depth interviews, HSA1 and HSA2 had this to say regarding the main tasks they perform in their facilities:

*“I am the health service administrator [HSA] here so I oversee to the day to day running of the facility and take care of properties within the facility. I make sure that logistics are available here for work and the facility has the required staff to take care of patients. I also compile reports to regional which is forwarded to the national level” (HSA 1 in CHAG* *facility K).*

*“As a qualified health administrator I was transferred to this place to manage a managerial crisis. This facility has lots of challenges and with my expertise the authorities thought I can help manage affairs. Currently apart from the paper works I undertake here such as signing procurement for essential items to be purchased, it is my responsibility to ensure that this facility meets the needs of clients by making sure that human resource issues are addressed and that the facility has adequate staff to function properly. In short I will say that I handle all administrative duties here and report to the regional level”. (HSA 2 in* *facility F).*

Some of the tasks described by PA6 (mentioned earlier), which include ensuring the availability of logistics and appropriate human resource, are some of the official tasks of the HSAs, as explained by HSA1 and HSA2 (above). It should be noted here that, they (the HSAs) are normally stationed at the districts and regional health facilities, and are not always physically present in the localities where the PAs work. This explains, in part, the growing practice of allowing the PAs to perform high level of responsibilities that were meant for the HSAs. However, some non-governmental organisations operate under a different structure that requires the HSA to perform their designated functions. For instance, the Christian Health Association of Ghana (CHAG) owned and run facilities have HSAs in some of the localities where they operate and should be seen to be performing their duties—though, they too, encourage some amount of delegation of responsibility to their PAs. By way of clarification, the CHAG facilities are also present where the GHS has their own facilities, such as in the rural communities. Collectively, these facilities, though run by different workers, are there to meet the health needs of the marginalised and vulnerable people and by so doing complementing the work of the Ghanaian Government’s Ministry of Health.

Another cadre of health staff trained basically to undertake preventive measures in rural communities are the Community Health Officers (CHOs). They carry out outreach programs in various schools in the communities within their sub-districts to give education on health care practices. Occasionally, they gather the community members to sensitize them on preventable diseases, such as Malaria and HIV/AIDS, and make home visits to pregnant and lactating mothers to advise them on the best health practices and the need to attend antenatal and postal care. They also render family planning services to clients in their respective health facilities and assist in running Child Welfare Clinics (CWC). Accordingly, CHO 2 and CHO 4 described the main tasks they perform in their facilities:

*“I and the other CHOs run the child welfare clinic in this facility. We also engage in family planning services and school health programs. We visit 27 schools to give health talk and also advice mothers with malnourished children on how to take care of them”.* *(CHO 2)*

*“I am supposed to provide assistance to the midwife during antenatal services, to conduct child welfare clinics, conduct defaulter tracing, I mean those who have defaulted from coming for their health care activities such as child welfare clinics and antenatal care. I also carry out daily routine home visits and community sensitization to educate community members on health care practices and preventable diseases”.* *(CHO 5)*

Apart from the main tasks performed by the different cadres of health workers, they sometimes perform additional tasks to keep the facilities running due to the inadequate staff members. Though their training generally put them in a position to be able to take up additional basic duties, such as checking temperature and blood pressure of a patient, respondents were of the opinion that sometimes they engage in tasks which they are not trained to do. Consultation of the sick is one of the extra duties performed by some staff members who said they acquired this skill through observations in their respective facilities and long service experience. It should be noted here that though staff members are aware of the possible implications of taking up tasks they have not officially been trained to do, they appear to be more committed in order to save the lives of patients for at least it is better than nothing. When a General Registered Nurse (GRN 2) and an Eye Specialist (ES 1) were asked during the in-depth interview whether they performed tasks which they were not officially trained to do, they confidently answered in the affirmative, but further explained that in spite of the risks associated with their additional workload, lives need to be saved and as such it is essential that they are engaged in the performance of these duties,

*“Oh yes I sometimes perform tasks which I am not officially trained for. There are somethings you are not supposed to do but you cannot say that you are not supposed to do that so patients should die because it’s a doctor’s duty. Even the consultation I am telling you, as a nurse I am not supposed to do that. Sometimes we take risk in taking care of the patients. If you mention this to any doctor they would tell you that nurses are not supposed to consult but we do. This is a rural area with no doctor here. If a baby is brought here running temperature, you need to get a doctor but you have to take care of that baby before later you refer to the appropriate places. Even in some places in the city, nurses are not supposed to pass cannulas but here we do. So these are some risky things we do”.* *(GRN 2)*

*“Yes we have to do that to keep the facility running. Am an eye specialist and ideally I should be treating only eye cases but I do general consultations. I stay in this cortex so most of the nights they come to wake me up to attend to patients. Medically I treat, I can do seizure very well and I can do circumcision very well. I just observed from other health personnel and now l can do it well. I don’t know how I obtain the skills to circumcise but I can do it very perfect. If I circumcise your child, you will like it. These are things we do here that we are not supposed to do but the specialist to do those tasks are not available. And at the end of the day, we do these tasks and somebody takes the credit because they don’t know that there are people in the rural areas doing such tasks. We do these things and others sit in the cities and take the glory. Nobody will appreciate you for the good work you are doing”. (ES 1, in one of the CHAG facilities, facility* *Y)*

As explained by ES 1, proximity to the health facility is also a compelling factor for staff members to take up additional tasks to save lives. In rural communities, where the road networks are bad, and ambulance service unavailable, the referral of patients, especially in the night hours, to the regional hospital is difficult, if not almost impossible. The concerns of ES 1 points to the fact that authorities (those at the top of the hierarchy) should be showing appreciation for the additional tasks they perform in these facilities but it has been difficult for authorities to show appreciation to health workers performing tasks they were not officially assigned to do. This is what a community health officer and a health aide has to say:

*“My tasks as a community health nurse are to go for outreach services, engage in child welfare clinics and visit people who were discharged from this health centre to check on their health to see if they are doing well. I sometimes do consultations but this is not part of my job because I have not been trained to do this. I always observe what goes on here and try to do something because you and I know that half a loaf is better than none so it’s better we do something then not doing anything at all. On the other side it’s dangerous because if I make a mistake of giving wrong drugs to a patient and she dies, I will be in serious trouble so am careful”. (CHO* *1)*

*“As a health aide worker, prescribing medicine for the sick is not part of my work here. But sometimes I do that. My main task is to assist in the OPD, assist the midwife in delivery, assists in the weighing of babies and other postnatal activities. The reason why I perform other duties is because I have worked in this health facility for a long time and I have experience in consultation. So when the in-charge is very busy or is not around, I do consultations because we have only one senior nurse here who is trained to be consulting”. (Nurse Aide* *1)*

Sometimes, some of the respondents assume the roles of their absentee colleagues, in addition to performing of their main tasks, so as to keep the facility running. It appeared respondents had no option than to take up these additional tasks, due to the critical situations they find themselves in within these facilities. For instance a CHO in the absence of a DCO and a midwife, take up their tasks. CHO 2 and GRN 3 explained the situation:

*“You know here we have only one disease control officer who has gone on leave. Because she is not around at the moment, am doing her work. I have added her work, my work and if midwife is not around, I will be forced to conduct deliveries and do ANC [Antenatal Care]. The staff is not enough and we are dealing with human life so if the people report, you can’t say you won’t help. At least you should try and do something to save the lives of the people”. (CHO* *2)*

*“I don’t like pretending because GHS [Ghana Health Service] is aware that we nurses consult here but they say we shouldn’t consult and they know that this health centre has only one medical assistant to attend to 100 to 200 patients a day. Can only the medical assistant do that? Of course no so consultation, prescribing, wound dressing; seizure is sometimes carried out by people like me. Am not supposed to refer patients but sometimes I do that. When I detain, I carry out my bedside nursing. Sometimes the midwife would call me to come and help. Am a nurse so am not trained to consult but I can’t leave people to die. The medical assistant is the administrator, the human resource manager and director here and how can only one person carry out all these tasks? Because am acting as the in-charge at the moment, am attending a program that is supposed to be attended by the PA if he was around. So in his absence, who do you think should consult”? (GRN* *3)*

Though some health care workers in the study areas perform additional tasks in order to minimise the effect of inadequate health workers in the study area, some of the tasks performed by these health workers were far beyond their actual job descriptions. Some of these tasks include the general cleaning of the facility, filling of insurance claims forms and fetching of water. Some respondents were of the opinion that it is an ineffective approach to human resource management and general hygiene. Because getting the health workers who are supposed to take care of patients to perform non-clinical tasks increase the workload. A General Registered Nurse (GRN 3) and DCO 2 had this to say doing the in-depth interviews:

*“As the head of the facility because I want work to go on, I have to do other things I am not supposed to do. Somethings when the taps are not flowing, I fetch water because nursing cannot go on without water and the boreholes are far from here. You will agree with me that this is poor management issues because how can a nurse who is supposed to take care of patients be doing things like this. We have complained to our superiors that we need a polytank [i.e. water storage] to store water so that when the taps are not flowing we can get water but nothing has been done about that. I have to sometimes fetch water from the borehole so that we can at least wash our hands and sometimes to sponge the babies with high temperatures”. (GRN 3 in-charge of facility* *K)*

*“If I tell you that I sometimes engage in the cleaning of the facility, I don’t think you will believe me but I do clean because we don’t have a cleaner in this facility and sometimes I have to fill the insurance claims forms. We have one auxiliary staff we call the nurse aide who normally cleans the facility but I cannot allow her to be doing the cleaning alone because that is not part of her official duties. She is supposed to assist the midwife during delivery, antenatal care and postnatal care services because sometimes the patronage is high such that the midwife cannot handle it alone. So just imagine that in the process of cleaning, you are called to attend to an emergency, you would not be able to wash your hands well because you will be in a hurry which is not hygienic”. (DCO* *2)*

Inasmuch as, some of these tasks are beyond their official duties, some reiterated that they are voluntarily engaged in non-clinical tasks such as cleaning and fetching of water to motivate the workers and make them feel appreciated in the work they do as described by Enrolled Nurse (EN 2) during the in-depth interview:

*“Sometimes I get up to do my cleaning here in the facility. Even though we have cleaners, to motivate them I help them to clean so that they would know that their services are needed. When there is no water, I pick up my bike to fetch for the facility so it’s good”. (EN* *2)*

Observably, this increased workload sometimes compromises the wellbeing of the patients—as some of these cleaning jobs are performed without proper sanitary procedures.

### Management of members’ perception on task-shifting

Discussions with management officials in the various health facilities show that because of the critical shortage of physicians and clinical personnel, task-shifting has become a common practice in these facilities. Management members highlighted some the benefits of task-shifting. According to them, this emergent practice of shifting roles and responsibilities lowers the cost involved in giving health workers additional training comparable to the traditional mode of training and could also help the health workers to identify their areas of expertise for future development. According to Key personnel involved in the human resource management of staff members thus,

*“The task shifting is a difficult situation but we sometimes have to improvise to keep the system running. We try to give these health workers necessary capacity boost before additional tasks are handed to them especially in consultations but I am not ruling out the possibility for untrained ones to be engaging in consultations and other tasks due to inadequate skilled personnel in some of these rural communities. But you know that the additional training though could be less expensive compared to training let say a nurse for 3years, it still involve money and therefore the need for financial commitment from the government. Sometimes too when staffs take up additional tasks, it helps us to identify their special areas of interest to support them develop their carriers in future. We are trying as much as we can to improve the situation”. (Key Person* *2)*

*“People would have to work more than they are supposed to do, sometimes not very happy but that’s the situation we have and we have to deal with it that way. We encourage them to do their best despite the challenges. The staffing is not bad but the problem is the different cadres of staff. We don’t have critical cadres of staff. For example in the area of dispensary technicians at the dispensary we don’t have enough staff and x-ray the whole region has only 3 technicians. This hospital has only 2 medical officers. We should have about 15 but we are even better because some have only 1. In the absence of the doctors we need physician assistants but we don’t have enough. For critical care nursing needed to run the system, we don’t have enough. But for general nurses its better. For example midwives though not enough it’s ok. It is normally the post basic courses programs, peri-operative, emergency care that we don’t have the people available” (Key Person* *1)*

*“For additional responsibilities I will say, yes! because everybody is doing more than they are supposed to do. The training is such that, everybody has knowledge in some aspects so mostly people voluntarily take up additional responsibilities. For instance we don’t have people trained in HIV or TB but some of the nurses still take up those responsibilities. Is somebody who is dedicated to duty who will do this but sometimes they expect some form of motivation because its additional work and should be appreciated in some form, either by getting an opportunity to go to school earlier than expected or in terms of monetary terms or given accommodation”. (Key Person* *3)*

From these quotations (above) we see that task-shifting is regarded as a way of training alternate health care workers or lay-persons to perform tasks generally considered to be within the scope of the medical profession [2,15]. Reference has been made earlier to the fact that, comparably task-shifting do help the health workers to identify their areas of expertise for future development in ways that the traditional model of training cannot ordinarily achieve.

From what have been said so far, it is evident that the policies governing the management of human resource for health, viewed from the politics surrounding its formulation, implementation and evaluation—including its associated consequences, is a more complex issue. And it is critical to the kind of tasks and expectations that Respondent (Key Person 1) was talking about. From a practical point of view, the success or failure of task-shifting, whether to complement the work of others or to fulfil a duty that no one is doing, depends on the policies that are in place, and the way how political thinking and the medical understanding of healthcare and service interact in the production of the conditions that make workers to be receptive to the idea of doing what they are not officially trained to do.

It is not just about the needs of the people that the workers are there to meet that matter alone in this regard. It is also there to serve a political purpose [43]. It is about making sure that the consequences do not work against the political establishment that case-manage the health sector. It is the theoretical understanding that, where rational actions are taken by workers, the aim is to maximize positive result-oriented outcomes and mitigate the negative outcomes [43,44]. However, formulating policies to ensure effective management of the task-shifting could be challenging due to bureaucratic regulation in the health system [45], and for that matter allowing an untrained health worker to perform tasks that requires official clinical training, does not meet standardize work ethics and as such the need to put in place appropriate policies to manage the work of health workers more effectively.

As a result of many factors—some political and others administrative, the nature and level of involvement of these junior health workers in the development, implementation and evaluation (to decide whether to continue or change) of policies is limited. Whilst the level of their involvement is partly determined by what is at stake, they hardly feel a sense of ownership in the processes. As such, some of these policies are not developed with an afterthought of its advantages (or disadvantage, thereof) to the personal development of the health worker. This argument begs further a question: would the involvement of junior staff lead to the maximisation of positive outcomes? Depending on the actors involved and the location of operation, answers to this also depends on how receptive the worker is to policies that are guiding his work, and how sensitive the policies are to career and personal development of the worker. The kind of sensitivity that is being referred to here is in line with the narrative of encouragement that Key Person 1 (quoted above) talked about, when he noted that “*We encourage them to do their best despite the challenges*. *The staffing is not bad but the problem is the different cadres of staff*. *We don’t have critical cadres of staff*.”

When understood within the context of health care worker retention, the worker must feel being appreciated—even if they tend to work more for less pay. But again, both respondents (quoted above) argued that the successes of the workers depended largely on level of interaction they have with human resources. Theoretically speaking [46], the interaction therefore between the worker and those that regulate what he/she is officially hired to do (as well as the performance of additional tasks) falls back to the question of whether the well-defined policy-oriented bureaucratic standards makes room for flexibility. Based on field observations, flexibility within the largely ineffective bureaucratic standards is called for. In Ghana, more generally, views about work and ethics have attracted varying interpretations—with of these narratives being an outcome of consideration to age, gender, education and location. As such, interviewing these health workers yielded so much information on the local health-related realities (which includes the many challenges these local facilities and the health workers are experiencing) in Northern Ghana.

### Health workers perceptions on task-shifting

From what have been explained so far, it is important to discuss the perceptions of health personnel regarding task-shifting, which is now a common practice in most of the health institutions in the rural communities in Northern Ghana. Earlier in the discussions, references were made about some of the official and additional tasks performed at the health facilities of these rural communities. But what should also be noted here is that perceptions about work are shaped by peer-influence. By peer-influence, one is referring to the impact that the whole (the team) has on the individual.

*“As a Physician assistant I do administrative work too such as appraising staff. Apart from that we sit down to know the needs of the facility so that as a leader I can delegate for them to purchase certain things as you heard me telling somebody to get syringes and needles from medical stores. I don’t have an administrator here so am a jack of all. I do disease surveillance at the sub-district level and report to the regional office. I also go for home visits to find out the challenges of the community members so that we can help to give them better services. As in-charge it is not my duty to do these things but I do them to keep the facility running. I have no specific roles because my services are demanded all the time. I cannot rest because I also attend to emergency services day and night but what is helping is that we work as a team so I sometimes delegate some of the tasks and make sure they are carried out properly by other staff. We help each other”. (PA 3 in facility* *K)*

*“Sometimes I do things like filling of claim forms, costing and other things so it’s difficult working here. In the absence of a staff, you would have to do everything as and when the need arises. Sometimes maternity would call you to assist. We encourage that kind of things so that we can help each other”. (DCO* *3)*

For most of the respondents, they are of the view that it is important that staff at health centres work as a team and seek to support each other to ensure that the work goes on and expectations are met. Emphatically, it is teamwork that sustains the practice of task-shifting in these local facilities. Accordingly, one Community Health Officer (CHO) and a Physician Assistant (PA) reiterated that,

*“I am a community health nurse but I engage in almost every activities here even filling of insurance claims form and sweeping the compound. That lady [making reference to the worker that interrupted our interview session] came to call me now because there is a labour case. The woman is full [as in being in labour] so I have to go and assist the midwife to do the delivery. In case the midwife was not around I am supposed to conduct the delivery. That is not part of my work but if she is not around, I have to do it. I have no option then to conduct the delivery. I have started writing a report which I have to finish and these forms on my table are insurance claims forms. My in-charge asked me to fill the forms so that the facility can claim the money from the insurance people. Apart from all these things, one of my routine tasks is to visit communities for outreach services and you know it is not easy because our roads are very bad especially during this time of heavy rains. Sometimes when the in-charge is very busy in the consulting room, I go there to assist. The Ghana Health Service also has routine programs such as polio immunization and child health promotion programs which I have to take part. As you can see, this is too much work for me but because we don’t have enough staff here, we have to support ourselves and work as a team so that we can save the lives of patients”. (CHO* *4)*

*“As an in-charge of this health facility, you know that there is nothing I can do to keep the facility running than to talk to the workers to take up additional tasks. When I am around, it’s not a problem because I can supervise them but the problem is when I am not here and the work also needs to go on. We have only one midwife in this health centre, if she is not around and I am not also here, the community health nurse is called to conduct deliveries especially when they report with head in vagina but honestly speaking a community health nurse is not supposed to conduct delivery alone without a midwife. The only thing I can say is that, we are managing to save lives”. (PA 2 in-charge of facility* *Y)*

Undoubtedly, CHO 4 acknowledges that the workload of a CHO is a tall order. But she is not the only one who faces this challenge. Indeed many other health workers also expressed this tall order regardless of the position of the individual health worker. The emphasis is also helping and supporting colleagues to ensure that patients are taken care of. This is clearly about the relationship existing between colleagues and the clients, and between them and the general society. Because, what promotes or hamper progress in a social context of workers’ perceptions on who get what and how is partly determined by the kind of peer-assistance a worker receives [47]. Theoretically speaking, this kind of thinking has been explained more extensively by rational choice scholars. For them, it is about creating a workforce whereby a worker will be able to find answers to the subjective questions of ‘what works for whom, in what circumstance and in what respects and how?’ [48,49]. The reason for saying this is that, the perception of the worker is like an ‘open system;’ a system that acknowledges that one’s connection to the other is prone to being affected by controlled and uncontrolled factors. Therefore, one cannot assume to be a part of a team that does not have its ups and downs. Work in itself is not a constant state of being and doing. It changes over time; it is subjected to a self-regulated and other-regulated cycle of interactive opinions and common reciprocal practices.

As the quoted statements of PA2 and CHO4 (above) indicates, apart from working in administration and disease surveillance within their sub-districts, their workload is extensive and this has negative impact on the perception on task-shifting. However, it is generally encouraging that some the health personnel in the targeted communities feel a sense of self-worth in doing more than expected. These health workers feel their dedication is not just about the difficulties they face in working in rural communities, but the opportunities of building their expertise and capacity for the future. By working in remote areas with fewer officials, they consider themselves to be dexterous and multipurpose health workers. This, according to some of the respondents, places them in a position to be functional wherever they may find themselves.

*“I feel good helping my colleagues. Ghana Health Service encourages team work so it’s good to help. Sometimes I go round to supervise what other people are doing when the in-charge is not around. This is not part of my usual work but when am on call, I do it. I see it as an opportunity to learn new skills. I don’t know where I will find myself tomorrow”. (EN* *1)*

*“I think they build you up. Though the workload might be too much and we are trained to do specific things, when you involve yourself in other tasks that you are not trained to do, you learn new skills and you will be able to help clients to survive since we do not have enough staff here. I am talking about the consultation and dressing of wounds because I am not trained to carry out those activities in this facility. If somebody gets an accident and is brought here you cannot say there is nobody here so you will not attend to the person. At least you have to do something to keep the person alive”. (Midwife 2 in facility* *X)*

Some of the ‘in-charges’ also regarded additional tasks as sense of good leadership. According to them, their ability to do more work by taking up additional tasks says a lot about their level of leadership. This could be due in part to the fact that, these ‘in-charges’ to a large extend are responsible for managing their facilities and reporting the progress to the regional directorate. Because they would be called to answer to queries regarding the issues that would arise in their facilities, they normally put in their best and are determined to go an extra mile irrespective of their ages just to sustain the health facilities. Whilst the motivation of the old in taking up more tasks was linked to their desires to leave behind good legacy, the young, is focussed on attaining higher heights in their carrier development as explained by these Physician Assistants.

*“I feel it’s my responsibility to do additional tasks because as a leader you don’t have to sit down to be served but you rather serve so I don’t see it as a bother. Once am alive I always ask for strength from the Lord which he would always give to keep me going. As a leader if you go strictly by your job description it doesn’t make you a good leader. You know you have to intervene in other areas so that together you can build up the place so that others can have confidence in you so that when you retire, people will still remember the good work you did”. (PA 4- a 50 year old in-charge of facility* *Y)*

*“When you put in a managerial position, it means that a lot is expected from you and so as the in-charge of a health facility, there is the need to do more to help your facility grow and by so doing, people would see you as a good leader who is willing to sacrifice for the people. I can also receive promotion when I work hard and through that develop my carrier fast”. (PA 1– a 33 year old in-charge of facility* *M)*

Also, within some of these rural communities, patients do find it difficult to distinguish between the workers within the facilities. During the field work, this was put to a test and the finding was that when called upon to distinguish between the different categories of health personnel most do make mistakes. Thus, it is difficult for patients to distinguish between a doctor and a nurse so they are of the perception that a nurse can do the job of a doctor or a physician. The patients addressing these health workers as doctors show their level of expectations. As far as these patients are concerned these health workers should be able to manage all cases in these rural facilities. According to these health workers, patients sometimes get angry and refuse referrals which in some cases worsen their health conditions and causes death. Therefore, in attempt to meet the expectations of clients, health workers are sometimes forced into handling additional tasks which they are not trained to do:

*“The people here don’t know that there are different nurses for different tasks. They know a nurse is a nurse so you should be able to deliver to make them happy. Sometimes they refer to nurses as doctors so if you are exposed to many tasks it helps you to meet the expectations of patients which will make you happy”. (Midwife* *4)*

*“There are antenatal mothers who come and routinely we need to monitor their HB [haemoglobin] and other things so we refer them to Bolga regional hospitals for scan and lab investigations. Others don’t go and their conditions worsen and sometimes it caused death because they would tell you they have no money for transport. That is why means of transport is very important. If there is transport available I can gather them and take a number say 5,6 or 10 for their HBs to be checked but there is no transport so we do what we can and allow God to take care of the rest”. (GRN* *4)*

Conversely, many of the health workers described the additional tasks they undertake as stressful and tiresome. They complained that they are over worked to the point that they are unable to go on leave, amid the fact that they are working in sub-sectors they do not have any skills in.

*“We are just overworking ourselves here. Yesterday I left here after 5pm and reported here very early this morning. I am hungry but there is no where you can go and take lunch or eat. And you can’t even go because the people have lined up out there waiting to be attended to. The year has almost ended and I have not taken my leave. I have to sacrifice for other staff to go on leave. If I don’t get my leave officially, I have to be a skeletal staff (on and off work) so I can get some time to rest. If I pick an official leave it means I have to stay away and if I apply for my leave now, it will run into next year which is not allowed so you can imagine what I am going through here”. (GRN 2 in-charge of the facility* *K)*

*“In fact it is very tiresome, one is always overstretched but you can’t do anything than to manage. Do you know that last night around 11:00pm I had two stressful labour cases? They were serious cases. Just when I was trying to figure out what to do to save the lives of these women, I was told there was an OPD case that needed immediate attention and this patient started shouting on me as if I was sitting down doing nothing. Meanwhile it is not my duty to handle that case. Tell me how one person can handle two different emergencies at the same time. This is too much work because you don’t even have time to take care of yourself but you know we have to always manage to push things through”. (Midwife 3 in facility* *M)*

The complaint associated with performing additional tasks could be due, in part, to the absence of incentives to motivate these health personnel. According to some of them, they are not given additional money to compensate their efforts.

*“In fact, it does not matter the number of additional tasks you perform, your salary is the same. They don’t give us anything for doing more work which is not good because our colleagues in the cities run shift so they can take up additional jobs to make more money but we cannot because this community is even too far from the city and we are on call 24 hours. At least they should give us some incentives to motivate us a little but there is nothing like that so you do more work but you do not receive anything apart from your salary which our colleagues in the cities also receive”. (Midwife 4 in facility* *L)*

*“It’s a mix feeling. Sometimes I feel privileged handling new tasks and sometimes too I feel stressed up because the workload is too much and no matter what you do your salary is the same. I close from work late and cannot even cook food to eat and I have to carry clinic work home to do but my salary remains same. It’s not easy. We need more staff to help us”. (GRN 3 in facility* *X)*

*“The only thing I can say is that, the workload is too much for me here. I am the administrator, accountant, and consultant and I also supervise the other staff so you can imagine what I am going through. There is no motivation for doing all these tasks but I am managing”. (Physician Assistant 4 in-charge of facility* *T)*

Some of the ‘in-charges’ of the various health facilities admitted that the additional tasks undertaken by staff members overburdened them but they manage to convince the health workers by encouraging them to be hopeful since there would be seasons when they would not have to do much.

*“What I see is that some of the staff think they are overburdened even me but what I use to console them is that, there would be instances where the workload would go down and during that time we could all have enough rest but that never happens because this is a busy place and so we have to sacrifice to keep the facility running. As the in-charge of the facility, I make sure that I regulate the times people go on leave so two nurses cannot both take their leaves at the same time”. (PA 6 in-charge of facility* *Y)*

Emphatically, and contrary to the pessimism of PA 6 (above), another health worker expresses optimism when she said;

*“I see it to be normal because we have to manage to run the facility because you are not doing it because your superiors want you to do those tasks but you are doing them because health workers to carry out those activities are not available but it’s very hectic because it at times takes you off your main duties. I am not supposed to officially prescribe but I sometimes do that because if you refer them to the next level, it’s always a problem because they need to carter for extra cost such as money for transport and other cost in the regional hospital. They sometimes refuse the referral and worsen their conditions or lose their lives. So you will agree with me that there is the need to ‘manage things’ here”. (EN 3 in facility* *F)*

The phrase “Manage things” (also dubbed “managing”) is a common phrase in Ghana. It means coping within extraordinary options with little or no innovative alternatives. Many health professionals in rural communities would use the word very often and with a small laugh to emphasize the sometimes-difficult situation beyond the control of the individual worker.

#### Health system and governance factors in implementing task-shifting

The organisation, structure and resourcing of health systems, are important issues that need serious consideration in the implementation of task-shifting [17] which is in accordance with the Realist Evaluation Framework approach [47]. Drawing from the concepts of the realist evaluation, the context and mechanism of a program are essential in determining it outcome. The mechanism, which is the process through which interventions are implemented, is very important because issues such as the target audience reaction and provision of adequate resources and capabilities determine whether a program would work or not. Therefore the mechanism regarding the practice of task delegation such as the health workers response, and adequate resources available for their training or for the provision of incentives to compensate these health workers for taking up additional tasks is crucial. This was explained by key person 4 during the in-depth interviews as follow:

*I believe if we have available accommodations and even the staffs are allowed to pay the cost, it would be good. The community cannot get for our staff members the type of buildings that can make them comfortable because of the economic status of the community members. It not easy because sometimes when you go out and see where people who are providing health care are living, the condition under which they are putting up is very sad and appalling. As a manager you have to encourage the person but the question you ask yourself is assuming that gentleman [referring to a health worker] is your brother or son and that lady your wife or sister how would you take that. So it is not easy at all! But we are trusting God for something better [it was observed during fieldwork that there is a connection between the way the worker feels at work and his/her religious belief]. The human element in service provision is very important. (Key* *Person 4)*

In a situation described by Key Personnel 4, it is not encouraging for health workers in rural communities, taking up more tasks than expected to have difficulties accessing suitable accommodation since this could affect the outcome of the intervention. However, to help address this challenge, efforts are been made by the management members to seek for financial assistance from the government and other private partners. Key Person 5 explained the effort underway in handling this:

*The ministry in collaboration with a private financier have approached as with a proposal entitled Build, Operate and Transfer (BOT). They would build, operate and transfer the buildings to us [for the health worker] later. Our new regional director has also started engaging other financial institutions and our suppliers from who we purchase the drugs, I mean the pharmaceutical companies to also assist because if you give us drugs and there are no human beings to administer the drugs, it can’t be feasible. Apart from accommodation, they have been asked to donate towards carrier development of the health workers. (Key Personnel* *5)*

From the narratives and discussions so far, clearly task-shifting has been the main stay in these rural facilities, where health workers are called upon to ‘manage’ to the best of their abilities and against all odds. This mechanism which was previously referred as substitution [15] has over the years gained international consideration. Over the decades, many countries have used this task-shifting as a mechanism to either respond to an emergency or as a means to provide adequate health care at primary and secondary levels more especially in understaffed rural facilities, to enhance healthcare quality and reduce cost [50]. However, there have been various arguments about the practicality, effectiveness and mode of operation since it was supported by the 2006 World Health Report.

The findings of the study show the importance of understanding the practicality and challenges associated with task shifting. Across the various health centres where the study was carried out, task-shifting was found to be a common practice and was practise among all cadres of staff though the extent of implementation varied from one health centre to another. It could lead to improvement in access to healthcare due to the teamwork approach employed by health workers and the sense of urgency they attach to support each other in taking care of clients in the health centres.

Teamwork is crucial for the successful implementation of task-shifting and supporting teamwork and supervision among health workers have been acknowledged largely to improve quality health care across the spectrum of health services [51–55]. It is also encouraging that health personnel are willing to engage in additional tasks to save lives of patients. Recent studies indicate that great urgency has recently been attached to task-shifting due to general health workers shortage to improve healthcare access [17,56]. The World Health Report of the WHO (2006) also advocates for the need to delegate tasks systematically to less specialised cadres of health workers to improve access to health care. Apart from health personnel building their expertise by engaging in new tasks, the task shifting practice could also lower cost in training health workers compared to traditional delivery methods of training [15,57]. However, training needs to be adequate and proper supervision put in place. Adequate supervision can also be achieved when some of these health workers are trained and released of some of their existing tasks to enable them supervise the additional tasks performed by other staff members [58]. This would not only lead to quality healthcare delivery but could lessen the workload of health workers who have taken up additional tasks.

Though adequate training and supervision are key in the implementation of task shifting to build staff competence and to ensure that quality healthcare is not compromised [2,19,59], these were observed to be generally lacking in the study area. Adequate training was usually not provided before additional tasks were assigned to staff members. Health workers as and when they are confronted with issues are called upon to manage to the best of their capabilities. Though this is in accordance with their expectations of learning on the job, this situation could lead to compromised healthcare and further lead to discontent and de-motivation since some personnel feel exploited rather than given the opportunity to learn.

It was also observed that in some cases the additional assigned tasks overburden staff and causes stress which could also lead to low motivation and defeat the purpose of the intervention. Research has established that too much stress due to increased workload on the part of health workers could lead to low motivation [14]. Perhaps one of the most common means of addressing the problem of low motivation is the use of incentives, commonly grouped as financial and non-financial incentives. There are many options that are available to policy makers beyond the provision of financial incentives. Studies have found that non-financial incentives, when implemented appropriately, are able to attract and retain health workers in rural and remote areas [60,61].

As discussed by management in the study area, achieving adequate training for health staff before additional tasks are handed to them require commitment beyond the provision of finance from the appropriate authorities. This is supported by findings from other studies which suggest that, for the successful implementation of task shifting, the organisation, structure and health services should be given the necessary consideration to enable the formulation of a suitable monitoring outline for training and building management capability [17,19,58]. However, this is not to say that additional tasks should not be accompanied with incentive packages to motivate staff members, because it is what brings food on the table. Findings from other studies suggest the need to provide appropriate incentive packages for additional tasks taken by health personnel as a form of compensation in task shifting practice [17,18,58]. But, not all work rendered can be paid for especially where the worker loves doing what he/she does, and where he/she is not expecting recognition or compensation for taking up additional tasks.

### Limitations

The limitations of this study need to be mentioned. The study used purposive sampling to select study participants for the in-depth interviews. The opinion of others may vary across regions and districts and so cannot be generalized to the other regions in the country. However, it has provided detailed and highly contextualized information and understanding on task-shifting practice which could inform policies. Also the study did not assess the quality of care provided by health workers during task-shifting practice and as such more research is needed to assess the quality of care provided over time during task-shifting practice.

## Conclusion

Our results suggest that task-shifting, although beneficial has some challenges. These challenges include lack of training before task is assigned, inappropriate allocation of task, lack of motivation and health worker burnout.

On the whole task shifting has the potential of contributing to addressing the impact of insufficient health workers in the study area, if only it is appropriately and systematically organised. It is important to provide systematic learning through regular modules on the job training of staff and to provide adequate supervision for staff to enhance their skills before additional tasks are handed to them so as to ensure the provision of quality health delivery. Also appropriate compensation mechanism in the form of incentive packages should be put in place to motivate health workers for the additional tasks they perform.

## Supporting information

S1 File Health workers interview guide. (DOCX)

S2 File Health managers interview guide. (DOCX)
