# Supplementary material for: Is task-shifting a solution to the health workers’ shortage in Northern Ghana?
Source: PLoS One. 2017 Mar 30;12(3):e0174631. doi: 10.1371/journal.pone.0174631 (PMC5373592; doi:10.1371/journal.pone.0174631)
Supplement: S1 File — (DOCX) [file pone.0174631.s001.docx]

**Health Workers Interview Guide on Task-shifting Practice**

(Expected time duration – 40 minutes)

**GENERAL INFORMATION**

1. Name of facility:
2. District:
3. Type of facility:
4. Time interview started:
5. Time interview ended:
6. Interview date:

**DEMOGRAPHIC CHARACTERISTICS OF RESPONDENT**

1. Respondent’s unique identification number :
2. Official designation:
3. Age (completed years):
4. Marital status:
5. Tribe:
6. Number of children:
7. Number of years worked in the facility:
8. Mode of posting to the health facility:

**Interview Comments:** This includes recording where the interview took place, mode of respondent during the interview, interactions and other non-verbal expressions of respondents that will help to understand the context of the interview.

**QUESTIONS/PROBES**

1. What are the official (main) tasks you perform in this facility?
2. Do you perform additional tasks which were originally performed by another staff? If yes, what are those tasks and what amount of your time is allocated to them?
3. Have you been trained officially to handle the additional tasks? If yes what type of training were you giving?
4. What is your general impression about the additional tasks assigned to you? ***If not mentioned, probe to find out about the strengths and challenges associated with performing additional tasks.***
5. Do you receive any form of incentive for performing additional tasks? If yes what are these incentives?
6. In what ways do you think task-shifting practice could be improved in this facility?

**For the heads of facilities (in-charge)**

1. **As the head of this facility**, how are you managing task-shifting practice in this facility?

**THANK YOU SO MUCH FOR YOUR TIME AND SUPPORT.**
