# Supplementary material for: Is task-shifting a solution to the health workers’ shortage in Northern Ghana?
Source: PLoS One. 2017 Mar 30;12(3):e0174631. doi: 10.1371/journal.pone.0174631 (PMC5373592; doi:10.1371/journal.pone.0174631)
Supplement: S2 File — (DOCX) [file pone.0174631.s002.docx]

**Health Managers Interview Guide on Task-shifting**

**(Expected time duration - 40minutes)**

**DEMOGRAPHIC CHARACTERISTICS OF RESPONDENT**

1. Respondent’s unique Identification number:
2. Official designation:
3. Age:
4. Sex:
5. Marital status:
6. Number of years worked:

**Interview Comments:** This includes recording where the interview took place, mode of respondent during the interview, interactions and other non-verbal expressions of respondents that will help to understand the context of the interview.

**QUESTIONS / PROBES**

1. How is task-shifting practice organised in the primary health care facilities? ***If not mentioned, probe to find out if health workers are officially trained before additional tasks are handed to them.***
2. In your opinion what would you say are the strengths associated with task-shifting practice?
3. What are the challenges associated with task-shifting practice?
4. What do you think could be done to improve task-shifting practice in these primary health care facilities?
5. Do you have incentive packages in place for health workers who take up additional tasks? If yes what are these incentives, if no why?

**THANK YOU FOR YOUR TIME AND SUPPORT.**
